# Insights into Ammonia Adaptation and Methanogenic Precursor Oxidation by Genome-Centric Analysis

**DOI:** 10.1021/acs.est.0c01945

**Published:** 2020-08-27

**Authors:** Miao Yan, Laura Treu, Xinyu Zhu, Hailin Tian, Arianna Basile, Ioannis A. Fotidis, Stefano Campanaro, Irini Angelidaki

**Affiliations:** † Department of Environmental Engineering, Technical University of Denmark, Bygningstorvet Bygning 115, DK-2800 Kongens Lyngby, Denmark; ‡ Department of Biology, University of Padova, Via U. Bassi 58/b, 35121 Padova, Italy; § School of Civil Engineering, Southeast University, 210096 Nanjing, China; ∥ NUS Environmental Research Institute, National University of Singapore, 1 Create Way, 138602, Singapore; ⊥ CRIBI Biotechnology Center, University of Padua, 35131 Padua, Italy

## Abstract

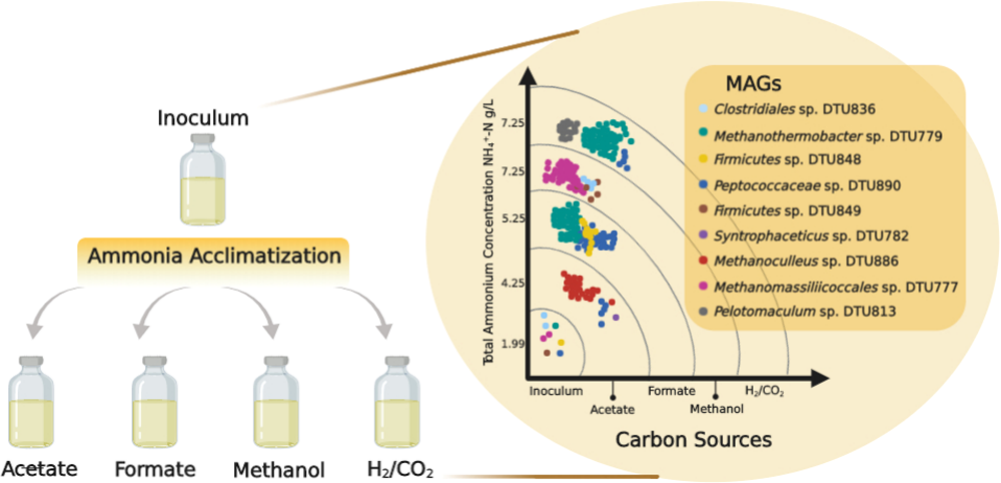

Ammonia released from the degradation of protein and/or urea usually leads to suboptimal anaerobic digestion (AD) when N-rich organic waste is used. However, the insights behind the differential ammonia tolerance of anaerobic microbiomes remain an enigma. In this study, the cultivation in synthetic medium with different carbon sources (acetate, methanol, formate, and H_2_/CO_2_) shaped a common initial inoculum into four unique ammonia-tolerant syntrophic populations. Specifically, various levels of ammonia tolerance were observed: consortia fed with methanol and H_2_/CO_2_ could grow at ammonia levels up to 7.25 g NH^+^-N/L, whereas the other two groups (formate and acetate) only thrived at 5.25 and 4.25 g NH^+^-N/L, respectively. Metabolic reconstruction highlighted that this divergent microbiome might be achieved by complementary metabolisms to maximize biomethane recovery from carbon sources, thus indicating the importance of the syntrophic community in the AD of N-rich substrates. Besides, sodium/proton antiporter operon, osmoprotectant/K^+^ regulator, and osmoprotectant synthesis operon may function as the main drivers of adaptation to the ammonia stress. Moreover, energy from the substrate-level phosphorylation and multiple energy-converting hydrogenases (*e.g.*, Ech and Eha) could aid methanogens to balance the energy request for anabolic activities and contribute to thriving when exposed to high ammonia levels.

## 1 Introduction

The amount of nitrogen-rich organic waste generated worldwide is increasing significantly because of urbanization and population growth, which is becoming a major issue for the environment.^1^ The application of anaerobic digestion (AD) can convert nitrogen-rich organic waste into a sustainable fuel.^2,3^ However, free ammonia nitrogen (FAN) released from the degradation of protein or urea, once exceeding the threshold concentration, is a key parameter leading to low methane yield and process instability in AD.^4^ Moreover, methanogens are more vulnerable to ammonia compared to the other AD microbes because of their weak cell wall structure lacking peptidoglycan.^5^ FAN that permeates into cells can be converted to ammonium by protonation,^6^ resulting in temporary proton imbalance, potassium deficiency, and strong osmotic stress.^7,8^ Therefore, K^+^ uptake is important for the microbial cells to overcome ammonia inhibition.^9,10^ Meanwhile, the synthesis or transport of osmoprotectants such as glutamate, glutamine, phosphate, N^ε^-acetyl-l-lysine, and glycine betaine has been reported to achieve osmotic balance and counteract ammonia inhibition.^10−12^ These compatible solutes allow the survival at high osmolarity and the colonization of ecological niches in environmental conditions.^13,14^ Therefore, more energy is needed for regulating the proton balance or potassium/osmoprotectant uptake during biosynthesis maintenance.^6,7^ The electron-bifurcating flavoprotein complexes in *Bacteria* contribute to energy conservation through the energy-converting reductase complex (Rnf) or the energy-converting ferredoxin-dependent hydrogenase complex (Ech).^15^ Specifically, Rnf catalyzes the reduction of NAD^+^ with ferredoxin, thereby conserving the free-energy change in an electrochemical proton potential.^16^ Likewise, HdrABC or Nuo present in methanogens is indicative of flavin-based electron bifurcation,^15^ which contributes to obtaining energy from methanogenesis.^17^ Additionally, Eha/b and Ech hydrogenases show high sequence similarity to the subunits of complex I, a protein pump, where they deposited NADH and reduced ferredoxin for the buildup of the proton motive force, suggesting an important role in adenosine triphosphate (ATP) synthesis.^18,19^ Finally, the energy compensation to maintain the cation- and osmobalance against ammonia stress can be obtained from substrate-level phosphorylation (Table 1). The more exergonic the reaction is, the higher ammonia level they can possibly tolerate.

**Table 1:** Standard Gibbs Free Energy of Relevant Reactions in the AD Process

reactions	Δ*G*°′ (kJ/reaction)	reference
4 methanol → 3CH_4_ + CO_2_ + 2H_2_O	–315	(20)
acetate → CH_4_ + HCO^–^ + H^+^	–36	(21)
4 formate + H^+^ + H_2_O → CH_4_ + 3HCO^–^	–130.4	(22)
4H_2_ + HCO^–^ + H^+^ → CH + 3HO	–135.6	(22)
acetate + HCO^–^ + H^+^ + 3H → propionate + 3HO	–76.1	(23)
4 methanol + 2CO_2_ → 3 acetate + 3H^+^ + 2H_2_O	–71	(24)

Different tolerance levels to the ammonia of AD microbiome have been previously observed; for example, anaerobic glucose degradation in batch reactors was inhibited by about 70% at 3.5 g NH^+^-N/L concentration and at a pH of 8.0.^25^ Yan *et al.* found that *Methanosaeta concilii* and *Methanosarcina soligelidi* were the dominant methanogens at low (less than 3 g NH^+^-N/L) and high ammonia levels (5–9 g NH^+^-N/L), respectively, when degrading municipal solid waste.^26^ Further, high ammonia levels suppressed acetoclastic methanogenesis and enhanced the hydrogenotrophic pathway, as evidenced by the increase of the relative abundance of *Methanoculleus* spp. co-digesting cattle slurry and microalgae.^4^ Westerholm *et al.* also discovered the strong impact of ammonia on the occurrence of syntrophic acetate-oxidizing bacteria and the increased abundance of hydrogenotrophic methanogens.^27^ The last was generally proved to be more resistant to ammonia than acetoclastic methanogens in many cases.^28^

However, all methanogens mentioned above mainly grow on acetate and/or CO_2_/H_2_, whereas the capability of ammonia tolerance of other methanogens dependent on methanol and formate (two other important precursors of methanogenesis) was infrequently reported.^29^ Obviously, the substrate, together with the concentrations of ammonia, could drive different complete and balanced microbiome formation, leading to variable capabilities of microbes to tolerate ammonia. Deciphering the metabolic pathway of ammonia-tolerant microbiome would improve our understanding of the dynamics and the molecular mechanisms determining stress resistance, which is necessary to unravel the black box of AD microbial ecology.

Until now, only part of the AD microbiome and its interactions have been uncovered because of the difficulty in exploring such complexity with traditional cultivation-based approaches and techniques (*e.g.*, 16S rRNA sequencing) because of the limited taxonomic assignment and the presence of unknown metabolisms. Metagenomics have been recently applied to analyze the known and novel physiological, metabolic, and genetic features.^16,30^ So far, most AD metagenomic studies focus on communities shaped by real feedstocks such as manure, wastewater, industrial by-products, and municipal solid waste containing various carbon sources.^31,32^ Accordingly, extremely diverse communities composed of thousands of metagenome-assembled genomes (MAGs) and complex metabolic activities adapted to mixed substrate degradation were found.^30,33,34^ These findings raise the possibility that specific interactions of ammonia-tolerant microbial members fed with single and simple carbon sources (the common precursors, *i.e.*, acetate, formate, H_2_–CO_2_, and methanol) and their functionalities await discovery.

This study provides novel insights into ammonia-tolerant methanogenic communities grown using four different carbon sources in a synthetic basal anaerobic (BA) medium. Specifically, a common initial microbiome was simplified with a stepwise increase of ammonia levels and meanwhile by providing single chemically defined substrates as an energy source: acetate, formate, H_2_–CO_2_, and methanol, individually. Genome-centric metagenomics was applied to unravel the methanogenesis pathways occurring in the four trophic groups. Moreover, the first look into the metabolism of the four microbiomes shaped by specific carbon sources showed how metabolic interactions occur among microbes at high ammonia levels.

## 2 Materials and Methods

### 2.1 Experimental Setup

The samples for microbial analysis were collected from four lab-scale methanogenic batch reactors with a 1.15 L total volume. The four reactors were initially inoculated with the same digestate obtained from a lab-scale continuous-stirring tank reactor fed with cattle manure at 55 °C. The total solids and volatile solids of the digestate were 30.51 ± 0.20 and 19.76 ± 1.30 g/kg, respectively. The feedstock used in each period was a BA medium^35^ (NaHCO_3_ was used as the buffer solution) supplemented with ammonia chloride and one of the four different carbon sources (acetate, methanol, formate, and H_2_/CO_2_), and thus the same buffering capacity was achieved (Table S1). Furthermore, the pH was maintained at the level of 8.00 ± 0.10 by NaOH solution (4 mol/L) adjustment throughout the whole acclimatization process (Table S1).

Several successive cultivations were performed under thermophilic conditions (55 ± 1 °C) in order to adapt the microbial community to the specific substrate and the increased ammonia levels. Specifically, once methane production reached 80% of its maximal theoretical yield during each generation, inocula samples were harvested to an increased ammonia level. The process was repeated in the four groups, and the ammonia level was increased stepwise by 1 g NH^+^-N/L in each increment until the microbial community could not grow anymore. The specific experimental conditions for consortia cultivation and acclimatization are listed in Table S1.

Methane yields, volatile fatty acid (VFA) concentrations, and pH values in the reactor were recorded in order to evaluate the acclimatization process. The biogas production was analyzed by a gas chromatograph (Mikrolab, Aarhus A/S, Denmark), equipped with a thermal conductivity detector. VFA concentrations derived from the intermediate steps of degradation of the carbon source were measured using a gas chromatograph (Shimadzu GC-2010 AF, Kyoto, Japan), equipped with a flame ionization detector. Finally, the pH was measured by a PHM99 LAB pH meter (RadiometerTM).

### 2.2 DNA Extraction and Sequencing

According to the specific carbon source used, the metagenomic DNA was collected from five sampling points: *G*_inocula_, an initial microbial community without additional ammonia and fed with cow manure (2.25 g NH^+^-N/L); *G*, a methanol-degrading community (7.25 g NH^+^-N/L); *G*, an acetate-degrading community (4.25 g NH^+^-N/L); *G*_formate_, a formate-degrading community (5.25 g NH^+^-N/L); and *G*, a H/CO-degrading community (7.25 g NH^+^-N/L) (Table S1). PowerSoil DNA Isolation Kit (QIAGEN, Germany) was used for genomic DNA extraction, and an additional phenol-cleaning step was performed in order to increase DNA purification.^36^ Nanodrop 2000 (ThermoFisher Scientific, Waltham, MA) was used to evaluate the quality of the extracted DNA.

### 2.3 Genome-Centric Metagenomics and Statistics

A sequencing strategy including both Illumina and Oxford Nanopore MinION single-molecule sequencers was chosen. Library preparation was performed using the Nextera DNA Flex Library Prep Kit (Illumina Inc., San Diego CA) and the SQK rapid sequencing kit (Oxford Nanopore Technologies, Oxford, UK); libraries were sequenced with the Illumina NextSeq 500 platform (Illumina Inc., San Diego CA) with a paired-end and FLO-MIN106 R9 flow cell on a MinION device (Oxford Nanopore Technologies, Oxford, UK) at the CRIBI Biotechnology Center sequencing facility (University of Padova, Italy). Raw sequences were uploaded to the Sequence Read Archive (NCBI) under the project PRJNA613371. Oxford Nanopore Technologies base-calling for translating raw electrical signals to nucleotide sequences was performed using Guppy (v2.3.7 + e041753).^37^ The total raw data provided 426,815,859 bases of sequence. Illumina reads with low-quality or ambiguous bases were filtered with Trimmomatic (v0.39). High-quality reads were independently assembled with three software, namely Spades (v3.13.0),^38^ OPERA-MS,^39^ and Unicycler (v0.4.8-beta).^37^ The assembly process was applied to Illumina reads alone and also to Illumina reads combined with Nanopore data using MEGAHIT (V1.2.4beta) software.^40^ After the assembly, all the scaffolds shorter than 1 kb were removed, and the statistics of the assemblies were determined using Quality Assessment Tool for Genome Assemblies (QUAST, V4.1).^41^ The scaffolds’ coverage was determined by aligning the reads of each sample back to the assembly with Bowtie 2 (v2.2.4)^42^ and converting the output SAM files to BAM format using SAMtools (v1.9).^43^ Metagenomic binning was performed using MetaWRAP software^45^ which implements Metabat2 (v2.12.1) and MaxBin2 (v2.2.6).^44^ Among the recovered MAGs, 143 were obtained from metaspades, 136 were from OPERA-MS, and 105 were from unicycler; the final selection was obtained by removing the redundancy and keeping the highest quality MAGs.

The completeness, contamination, and genome properties of the final MAGs were determined using CheckM (v1.0.3), and details can be found in Table S2. The relative abundance of microbes on each sample was obtained by aligning the reads to the assembly and subsequently using “BAM” files to calculate the final values using CheckM coverage (v1.0.3).The diversity index for each sample was measured from the unassembled Illumina reads using Nonpareil v3.303 with default parameters.^45^

Similarity with publicly available genomes was calculated by means of average nucleotide identity (ANI),^46^ and the results are reported in Table S3. Taxonomical assignment and functional analysis were performed using GTDB-Tk^47^ and CAT.^48^ Protein-encoding genes were predicted using Prodigal (v2.6.2)^49^ run in normal mode and associated with KEGG IDs using Diamond (v0.9.22.123).^50^ The KEGG IDs were associated with modules to determine completeness using the KEGG mapper-reconstruct pathway tool, as previously described.^51^ The functional visualization of MAG metabolism was performed using GhostKOALA.^52^ Hierarchical clustering of the binned MAGs across five samples was constructed using the MultiExperiment viewer (v4.9.0) with the Pearson distance metric and visualized by Anvi’o.^53^

Simultaneously, MAGs were used for genome-scale metabolic reconstruction and the subsequent analysis of interactions within a flux balance analysis framework, adopting CarveMe (v. 1.2.1)^54^ for the genome-scale metabolic reconstruction and a revised version of MMinte software (v.1.0.3)^55^ for the inspection of interactions, following the pipeline developed by Basile and colleagues (https://github.com/arianccbasile/ADinteractions). Literature-guided metabolic reconstruction was manually performed based on the genes and pathways present in the most abundant MAGs for each microbiome.

Four genomes of ammonia-sensitive *Methanosaeta* spp. were downloaded from public databases of NCBI to compare the energy-converting mechanism. The GenBank assembly accession numbers of these four genomes were GCA_012729025.1; GCA_012798255.1; GCA_012798025.1; and GCA_012516895.1.

## 3 Results and Discussion

### 3.1 Ammonia-Tolerant Reactor Performance Using Different Carbon Sources

**Figure 1:**
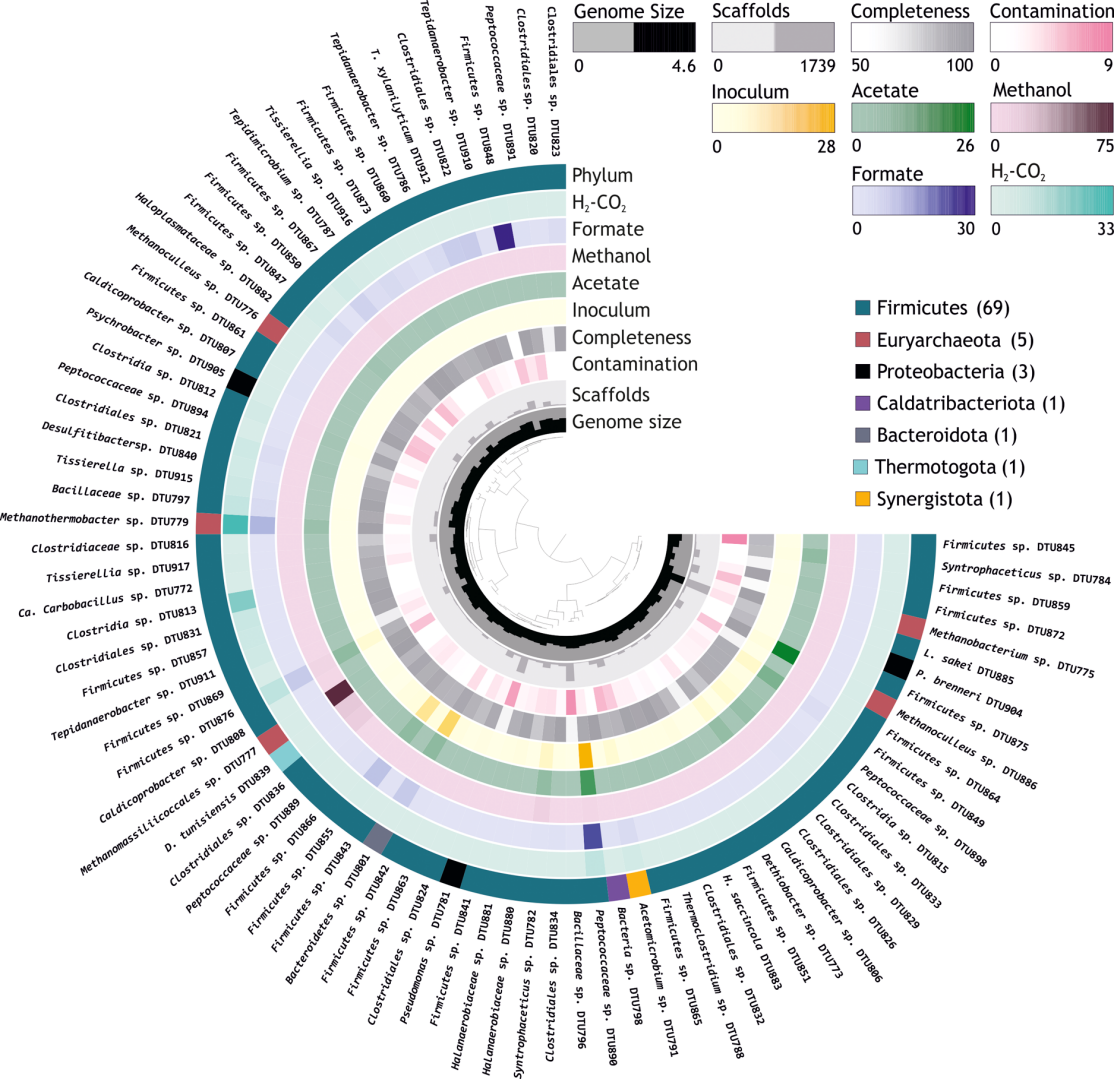
Microbial samples collected from five points of the batch reactors: *G*_methanol_, *G*_formate_, *G*_inoculum_, *G*_acetate_, and *G*_H_2_/CO_2__. The characteristics (coverage, quality, and taxonomic assignment) of 81 MAGs comprising the microbiome are reported. The outer layer represents the taxonomy at the phylum level. The five middle layers report the relative abundance of each MAG in the different microbiomes (% of relative abundance). The completeness (%), contamination (%), number of scaffolds, and genome size (Mbp) are colored in green, red, gray, and black, respectively. The middle phylogenetic tree represents the Pearson clustering of MAGs based on the relative abundances.

**Figure 2:**
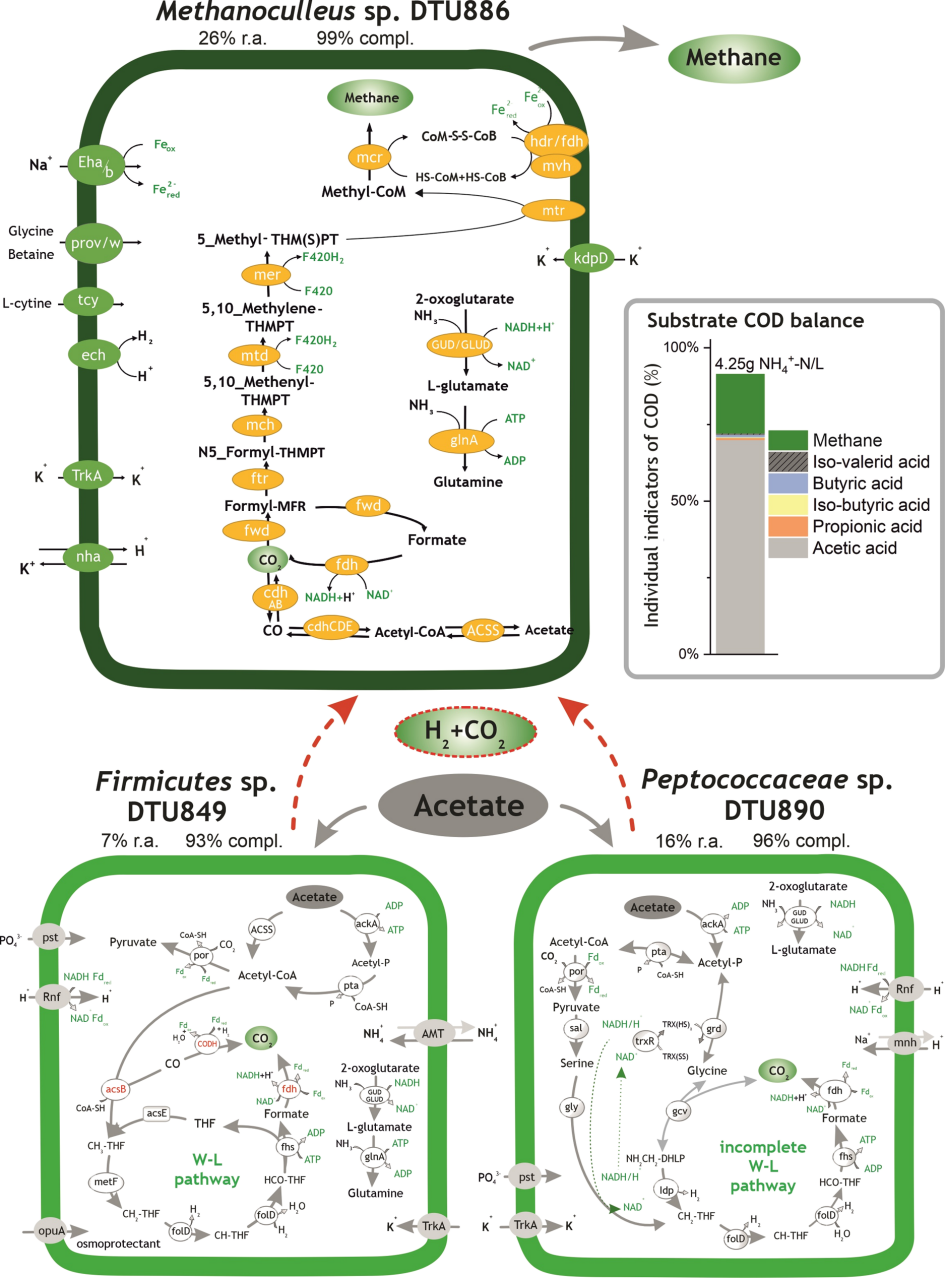
Histogram on the right side represents the substrate digestion profile (COD flow) measured in *G*_acetate_. Obligate syntrophic acetate degradation pathway proposed in *Methanoculleus* sp. DTU886, *Firmicutes* sp. DTU849, and *Peptococcaceae* sp. DTU890. “R.a.” and “compl.” are abbreviations of the terms “relative abundance” and “completeness”, respectively. The red dotted arrows represent the syntrophic intake of H_2_/CO_2_ by methanogens. All the relevant genes used for metabolic reconstruction can be found in Table S6.

**Figure 3:**
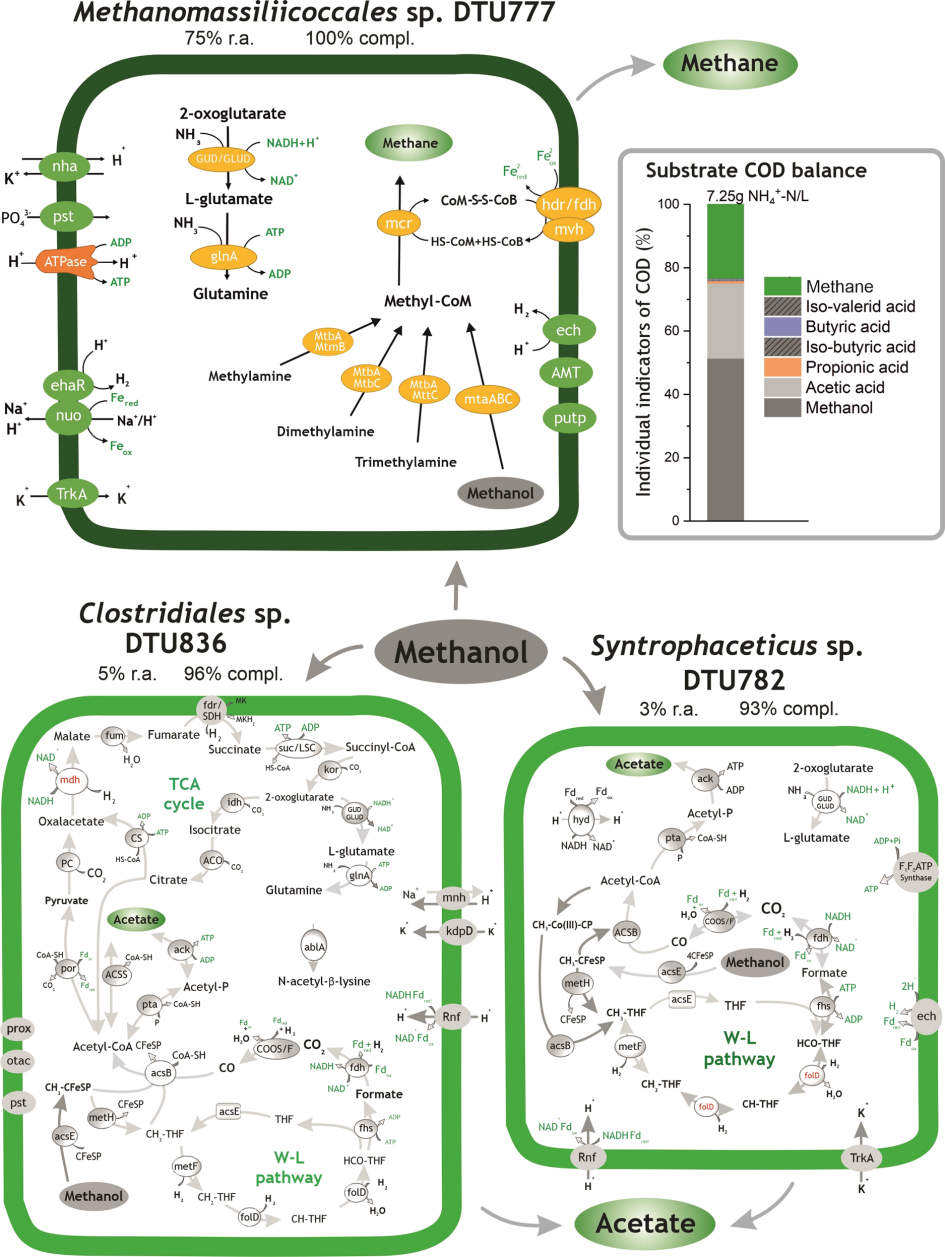
Histogram on the right side represents the substrate digestion profile (COD flow) measured in *G*_methanol_. Methanol degradation pathways identified in *Methanomassiliicoccales* sp. DTU777, *Syntrophaceticus* sp. DTU782, and *Clostridiales* sp. DTU836. “R.a.” and “compl.” are abbreviations of the terms “relative abundance” and “completeness”, respectively. All the relevant genes used for metabolic reconstruction can be found in Table S6.

**Figure 4:**
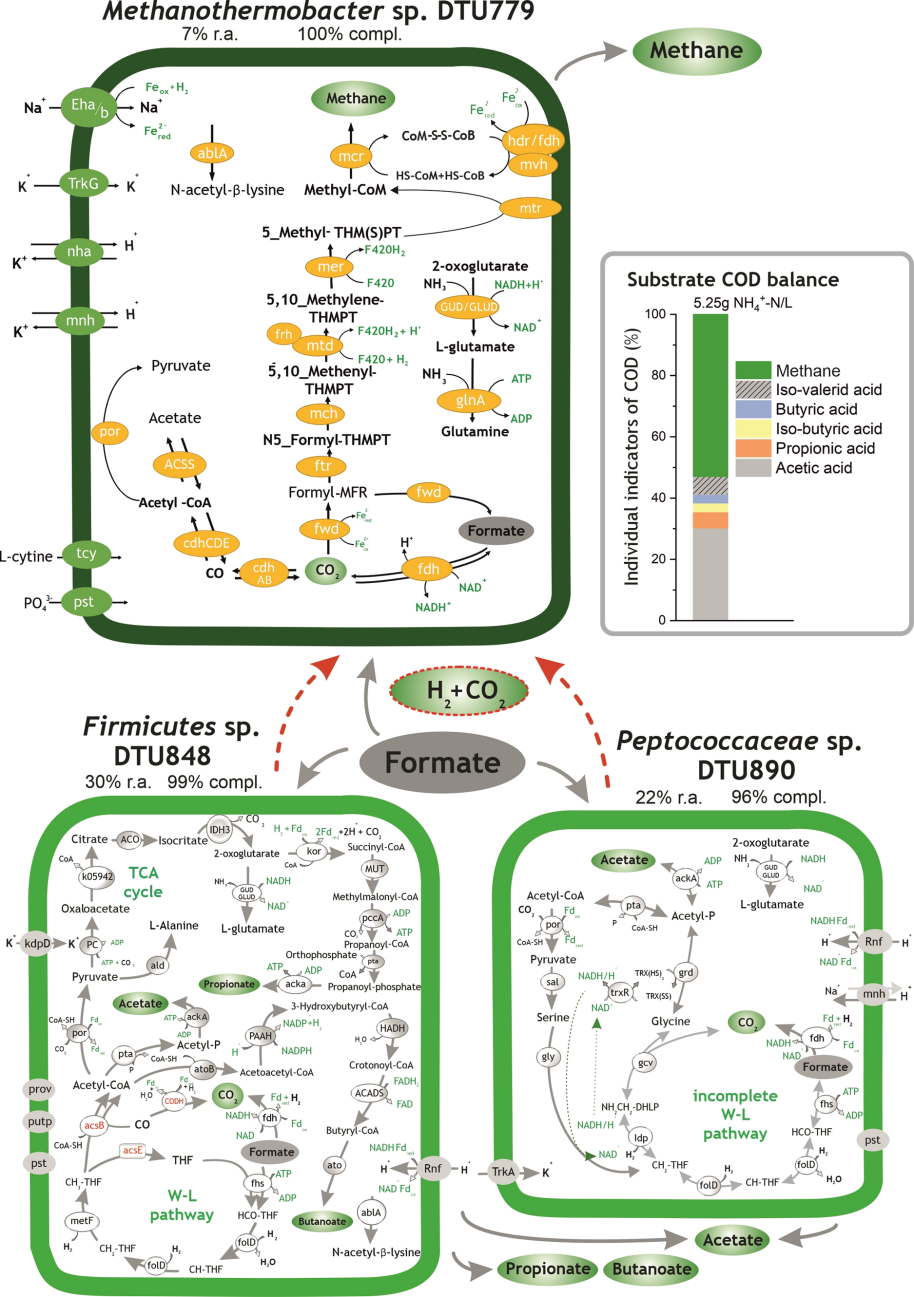
Histogram on the right side represents the substrate digestion profile (COD flow) measured in *G*_formate_. Formate degradation pathways identified in *Methanothermobacter* sp. DTU779, *Peptococcaceae* sp. DTU890, and *Firmicutes* sp. DTU848. “R.a.” and “compl.” are the abbreviations of the terms “relative abundance” and “completeness”, respectively. The red dotted arrows represent the syntrophic intake of H_2_/CO_2_ by *Bacteria*. All the relevant genes used for metabolic reconstruction can be found in Table S6.

**Figure 5:**
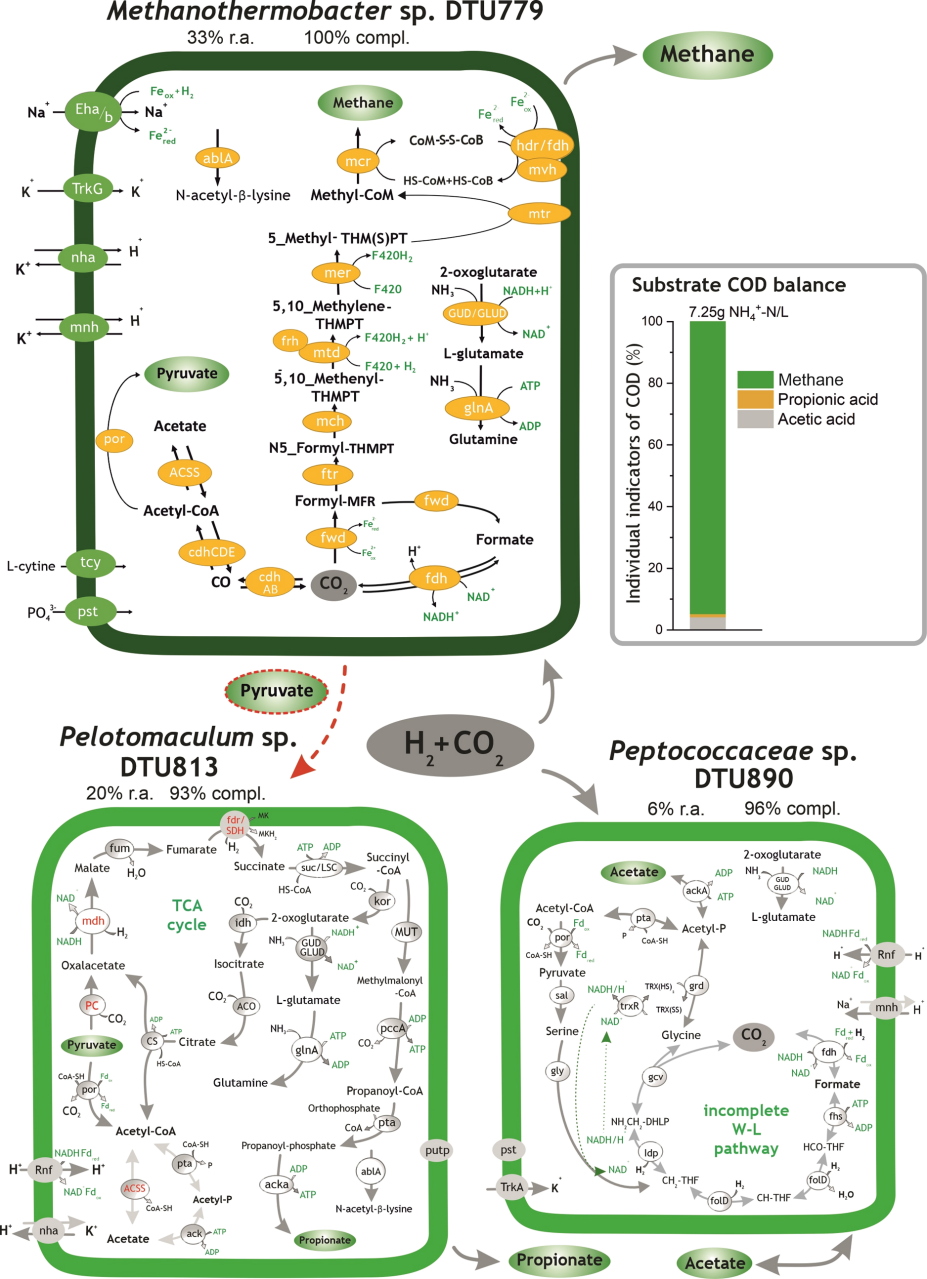
Histogram on the right side represents the substrate digestion profile (COD flow) measured in *G*_H_2_/CO_2__. H_2_/CO_2_ degradation pathways identified in *Methanothermobacter* sp. DTU779, *Peptococcaceae* sp. DTU890, and *Pelotomaculum* sp. DTU813. “R.a.” and “compl.” are the abbreviations of the terms “relative abundance” and “completeness”, respectively. The red dotted arrows represent the syntrophic intake of pyruvate by methanogens. All the relevant genes used for metabolic reconstruction can be found in Table S6.

The microbial consortia were cultivated in batch reactors fed with specific carbon sources, namely acetate, methanol, formate, and H_2_/CO_2_. After five to six consecutive generations of cultivation under stepwise ammonia increase, the microbial species present in each group showed different capabilities of resistance to ammonia (Figures 1–5). Specifically, in comparison to other groups, the community in *G*_methanol_ and *G*_H_2_/CO_2__ showed higher resistance to ammonia inhibition and were able to grow up to 7.25 g NH^+^-N/L. Additionally, the highest methane yield (up to 91%) could be observed in *G*_H_2_/CO_2__ at 7.25 g NH^+^-N/L (Figure 5). On the contrary, the lowest methane yield (19%) was found in *G*_acetate_ at 4.25 g NH^+^-N/L (Figure 2). VFAs (*i.e.* acetate, propionate, iso-butyrate, butyrate, and iso-valerate) were detected as an important indicator of chemical oxygen demand (COD) flow from the substrate during the metabolic degradation driven by the microbial community. Trace amounts of VFAs were present in *G*_acetate_ and *G*_H_2_/CO_2__ (Figures 2 and 5); more than 20% of acetate (COD ratio of acetate to the added carbon source) was found in *G*_methanol_ and *G*_formate_, suggesting that acetate is a key intermediate during carbon degradation at high ammonia levels. To clearly decipher the main metabolic pathways that occurred during the different substrate degradations, the methane production and the intermediate accumulation (*e.g.*, VFAs) were expressed as the percentage (%) of the overall COD content to highlight the transformation processes and directly couple them with the metagenomic data. Besides, the methane yield is reported in Figure S1.

### 3.2 Microbial Community Composition and Activities

The assembly and binning process resulted in a total of 81 MAGs based on sequence mapping, and these microbial species accounted for 62.5–91.8% of the entire community, depending on the sample (Table S2 and Table S4). These MAGs represented the most abundant members of the microbiome; 52 out of 81 MAGs were of high quality (more than 90% completeness and lower than 5% contamination), whereas the remaining 29 MAGs were of medium quality (completeness from 50 to 90% and contamination from 5 to 10%) according to the minimum information about the metagenome-assembled genome (MIMAG)^56^ (Figure 1 and Table S4). The 81 MAGs were taxonomically assigned into seven phyla, namely *Firmicutes*, *Proteobacteria*, *Thermotogae*, *Actinobacteria*, *Chloroflexi*, *Bacteroidetes*, and *Euryarchaeota* (Figure 1).

The different carbon sources (methanol, formate, acetate, and H_2_/CO_2_) used in this study, as well as the stepwise increased ammonia levels, worked as selecting pressure that shaped the microbial communities inducing considerable distinction in terms of diversity. In particular, attention was focused on the dominant members in each microbiome (Figures 1–5) and on the corresponding metabolic maps that were reconstructed using KEGG modules (Table S5), as well as individual gene’s annotation and literature-based information. Hydrogenotrophic methanogenesis using H_2_ or formate as electron donors was observed as the only methane-producing pathway in *G*_formate_, *G*_H_2_/CO_2__, and *G*_acetate_. Meanwhile, methylotrophic methanogenesis was solely observed in *G*_methanol_, as revealed by the presence of methanol transferase and methyl-CoM reductase in *Methanomassiliicoccales* sp. DTU777.

The presence of acetate in each reactor was indicative of acetogenesis, performed *via* the conventional Wood–Ljungdahl (WL) pathway, methanol oxidation, and glycine cleavage system (Figure 3 and Table S6). The assumption is evidenced by the presence of these pathways in *Firmicutes* sp. DTU848, *Clostridiales* sp. DTU836, and *Peptococcaceae* sp. DTU890. In addition, a novel propionate synthesis pathway was reconstructed in *Firmicutes* sp. DTU848 based on the gene presence (*e.g.*, k05942, *kor*, and *acka*) using KEGG and the analysis of residual metabolites present in the medium (*e.g.*, acetate, propionate, and methane), growing in *G*_formate_ (Figure 4 and Table S6). The following sections focus on how interspecies interactions were established in a syntrophic consortium and the resistance mechanism to high ammonia levels.

#### 3.2.1 Microbiome in the Original Inoculum

In *G*_inocula_, *Bacteria* dominated the microbial community with a relative abundance of 98% of the binned microbiome (this value refers to the percent of reads aligned on the binned scaffolds), which accounted for 63.1% of the entire community, whereas *Archaea* were quite rare. Specifically, sugar-converting microbes (identified by the presence of Embden–Meyerhof pathway) represented the dominant MAGs consisting of *Peptococcaceae* sp. DTU890, *Bacteroidetes* sp. DTU801, *Firmicutes* sp. DTU855, and *Firmicutes* sp. DTU849 with 27.8, 16.6 10.7, and 3.8% of relative abundance, respectively (Figure 1 and Table S5). Results from ANI evaluation indicated that *Peptococcaceae* sp. DTU890 was 99.8% similar to *Clostridiales* sp. DTU010 and to other MAGs previously identified in different AD systems (Table S3).^57^ According to the pathways present in these *Bacteria*, they are capable of performing a complete fermentation, converting glucose to acetate *via* the Embden–Meyerhof pathway and pyruvate oxidation (Table S5). These results show consistency with previous findings which suggested the main driving forces expanding the complexity and stability of the AD microbiome.^58^ Meanwhile, *Peptococcaceae* sp. DTU890 was also involved in acetogenesis using a novel glycine cleavage system and the phosphate acetyltransferase–acetate kinase pathway (Figure 3). The other two MAGs, namely, *Syntrophaceticus* sp. DTU782 (4.3%) and *Acetomicrobium* sp. DTU791 (2.9%), were predicted to show the acetate-oxidizing ability that may work in syntrophy with hydrogenotrophic methanogens for methane production. The five identified *Euryarchaeota* sp. represented only 1.28% of the whole microbial community; among these, the most dominant MAG was *Methanoculleus* sp. DTU886 with 1.2% of relative abundance, followed by *Methanothermobacter* sp. DTU779 and *Methanomassiliicoccales* sp. DTU777, with 0.05 and 0.03%, respectively. Methanogenesis was performed by these three archaeal MAGs, having different metabolic traits of performing hydrogenotrophic and methylotrophic methanogenesis. Interestingly, no acetoclastic methanogens have been identified in the initial inoculum. The possible explanation is that the total ammonia level in *G*_inocula_ was 2.25 g NH^+^-N/L, which possibly suppressed the abundance of acetoclastic methanogens. This result also agrees with the microbial community of inocula (collected not on the same day but under the same operating conditions with our initial inocula) analyzed by 16S sRNA gene amplicon sequencing in our previous research.^59^

#### 3.2.2 Ammonia-Tolerant Microbiome in the Acetate-Based Medium

In *G*_acetate_, the microbiome shifted markedly, as evidenced by the change in the relative abundance of dominant MAGs when compared with the initial inoculum. In fact, the population evolved into a more simplified and specialized community, as confirmed by the diversity indexes (Table S7). *G*_acetate_ was dominated by *Methanoculleus* sp. DTU886, *Firmicutes* sp. DTU849, and *Peptococcaceae* sp. DTU890 (Figure 2), with a cumulative relative abundance of 48% (Table S4).

More specifically, *Methanoculleus* sp. DTU886 had 99.6% ANI when compared with Candidatus *Methanoculleus thermohydrogenotrophicum*.^60^ The archaeon dominated the microbiome with 26% of relative abundance and was the only methanogen present in the community. It was previously reported that *Methanoculleus* sp. could perform methanogenesis from H_2_/CO_2_ or formate but not acetate.^61^ Interestingly, *Methanoculleus* sp. DTU886 in this study was found to harbor a series of genes for the conversion of acetate to CH_4_ as well as the genes for H_2_/CO_2_ oxidation to CH_4_ (Figure 2). Furthermore, the genomes of *Firmicutes* sp. DTU849 (7%) and *Peptococcaceae* sp. DTU890 (16%) encoded proteins involved in H_2_ and CO_2_ generation, suggesting the presence of a syntrophic interaction occurring between these two species and the methanogen. The presence of such interplay was confirmed by flux balance analysis revealing that *Methanoculleus* sp. DTU886 is favored by the interaction within both couples (Table S8). Specifically, *Firmicutes* sp. DTU849 possesses an incomplete gene set involved in the conventional syntrophic acetate oxidation pathway for H_2_/CO_2_ generation through the reverse WL pathway, whereas *CODH*, *acsB*, and *fdh* were not identified. According to the reconstructed pathway, acetate was possibly converted to pyruvate through the inverse phosphotransacetylase–acetate kinase pathway and acyl-CoA synthetase pathway (ACS). The genes encoded in *Peptococcaceae* sp. DTU890 suggested the use of an alternative glycine cleavage system for acetate oxidation (Figure 2). Specifically, the glycine cleavage system was combined with a partial WL pathway to convert acetate to CO_2_/H_2_, supporting the syntrophic activity with hydrogenotrophic methanogens.^62^ Both syntrophic *Bacteria* possess the Rnf complex, which is involved in proton motive force-driven reverse electron transport from NADH to Fd_ox_, where Fd_red_ was produced as a high-energy-electron carrier to facilitate H_2_ generation. Regarding energy metabolism, *Methanoculleus* sp. DTU886 encodes a set of energy-conserving hydrogenases (Eha/b, Ech, and Fdh) contributing to the proton motive force by coupling proton translocation across the membrane to Fe_red_; the same set of proteins can also be used for CO_2_ reduction (Figure 2). Furthermore, methyl-THMPT HS-COM methyltransferase (Mtr), the membrane-bound enzyme complex, extruded Na^+^/H^+^ out of the cell, creating a Na^+^/H^+^-based ion motive force used for both ATP generation and methanogenesis.

#### 3.2.3 Ammonia-Tolerant Microbiome in the Methanol-Based Medium

In *G*_methanol_, the dominant *Methanomassiliicoccales* sp. DTU777 (75% of relative abundance) was the main player that was responsible for methane generation from methanol (Figure 3). The complete methanogenic pathway from methanol and methylamine was found in the genome (Figure 3 and Table S6). Additionally, the presence of membrane-bound NADH-ubiquinone oxidoreductase (Nuo) suggested the formation of a Fpo-like complex, capable of reoxidizing the reduced ferredoxin, with the concomitant translocation of protons or sodium ions across the membrane (Figure 3 and Table S6). The proton gradient generated by the complex mentioned above facilitated the ATP synthesis, employing the energy-conserving hydrogenase (Ech) complex, thereby coupling methane generation with energy conservation and enabling internal hydrogen cycling.^12,63^

*D. tunisiensis* DTU839, *Syntrophaceticus* sp. DTU782, and *Clostridiales* sp. DTU836 accounted for 7.3, 3, and 5% of relative abundance, respectively, and *Syntrophaceticus* sp. DTU782 and *Clostridiales* sp. DTU836 were chosen as the representatives of the whole bacterial community because of their high genome completeness and relative abundance. The flux balance analysis revealed a parasitic interaction between these two microbes, with *Clostridiales* sp. DTU836 taking advantage of the coexistence with *Syntrophaceticus* sp. DTU782 (Table S8). The presence of acetate in *G*_methanol_ suggested that acetogenic methanol degradation was performed as reported in the following description. According to the metabolic reconstruction, the methylic group was probably transferred to the methyl acceptor––corrinoid Fe–S protein (CFeSP) into CH_3_–CFeSP––and followed two possible pathways of CH_3_–CFeSP oxidation. First, a part of CH_3_–CFeSP was converted into acetate *via* the acetate kinase pathway; second, the rest of CH_3_–CFeSP was oxidized through the WL pathway, with a concomitant reduction of CO_2_ into acetate. The reduction of ferredoxin and ATP for energy conservation for the above two pathways would occur following previously proposed mechanisms.^24,64^*Syntrophaceticus* sp. DTU782 had the potential to perform the first pathway using methyltransferase and acetyl synthase (ACSE and ACSB); these genes can activate and transfer the methyl group to a corrinoid Fe–S protein and oxidize it to acetyl-CoA *via* ACSB (Figure 3 and Table S6).

Meanwhile, *Clostridiales* sp. DTU836 harbored the two complete gene complexes related to acetate generation from methanol (Figure 3 and Table S6). The excess of ATP derived from the first pathway (the oxidation of one methanol to acetate) might be sacrificed to drive the endergonic oxidation of 2-methyltetrahydrofuran to 5,10-methylenetetrahydrofolate, which is in consistence with the previous study.^64^

#### 3.2.4 Ammonia-Tolerant Microbiome in the Formate-Based Medium

The microbiome of *G*_formate_ was mainly composed of two highly abundant *Bacteria*, *Peptococcaceae* sp. DTU890 and *Firmicutes* sp. DTU848, and one *Archaea*, *Methanothermobacter* sp. DTU779, with an aggregate relative abundance of 59% (Figure 5 and Table S4). The analyses of flux balance revealed that the growth rate of *Methanotermobacter* sp. DTU779 is positively influenced by the presence of *Firmicutes* sp. DTU848 (Table S8).

In *Methanothermobacter* sp. DTU779, the reduction of CO_2_ to formyl-MFR using H_2_ was driven by the electrochemical sodium ion potential (Nha and Mnh) (Figure 4 and Table S6). Furthermore, methyl-COM reduction to methane could proceed *via* the MvhADG/HdrABC complex and was coupled to ferredoxin (Fd) reduction.^65,66^ Two sets of energy-conserving hydrogenases, Eha and Ehb, were found in the genome of *Methanothermobacter* sp. DTU779 (Table S6). These genes were shown to be critical for the refilling of methanogenesis intermediates (*e.g.*, H_2_) and for CO_2_ assimilation.^17^*Firmicutes* sp. DTU848 harbored the genes involved in the conversion of formate to acetate *via* the partial reverse WL pathway, which can explain the presence of acetate in *G*_formate_. According to the metabolic reconstruction, propionate could be generated through a novel pathway, which involves the oxidation of acetyl-CoA into pyruvate *via* pyruvate ferredoxin oxidoreductase and the final step of amination to form citrate. Similarly, isocitrate could be transformed from citrate catalyzed into oxoglutarate, then oxidized into succinyl-CoA, and further into methylmalonyl-CoA (Figure 4 and Table S6). Finally, methylmalonyl-CoA can be converted into propionate *via* propionyl-CoA carboxylase and phosphate acetyltransferase, as previously described by Bar-Even *et al.*(67) Thereby, the sodium pumping pathway coupled with the decarboxylation of methylmalonyl-CoA derived from succinate-CoA to propionyl-CoA with the pumping of two Na^+^ across the cell membrane, leading to a net energy gain.^68^ Therefore, the clear carbon flow from formate conversion to propionate generation and the reductive citric acid (rTCA) cycle found in *Firmicutes* sp. DTU848 for energy conservation^69^ confirmed that the bacterium could outcompete *Methanothermobacter* sp. DTU779 (30–7% of relative abundance) for formate utilization. As mentioned before, the absence of acetyl-CoA synthetase in the genome of *Peptococcaceae* sp. DTU890 indicated that an alternative glycine cleavage system was possibly employed for acetate oxidation. Additionally, both *Peptococcaceae* sp. DTU890 and *Firmicutes* sp. DTU848 encoded a sodium-ion pump (Rnf) that coupled the electron transfer for H_2_ generation and ATP synthesis (Figure 4 and Table S6).

#### 3.2.5 Ammonia-Tolerant Microbiome in the H_2_/CO_2_-Based Medium

In the *G*_H_2_/CO_2__ microbiome adapted to 7.25 g NH^+^-N/L, *Methanothermobacter* sp. DTU779 reached a remarkable relative abundance of 33% (5 times more than that in *G*_formate_) (Figure 5). This finding indicated that, in the presence of formate, hydrogenotrophic methanogenesis was the main pathway. However, it can be assumed from these results that *Methanothermobacter* sp. DTU779 prefers H_2_ as an electron donor for autotrophic growth, when compared to formate. Additionally, according to similarity results, DTU779 was found to have 99.8% of ANI with *Methanothermobacter wolfeii* and with other MAGs identified in previous studies^70,71^ (Table S3). Interestingly, it is evidenced by Lins *et al.* that by replacing formate with H_2_ in the feed, the doubling time of *M. wolfeii* can decrease to 7.65 h.^72^

The bacterial community in *G*_H_2_/CO_2__ is mainly represented by *Pelotomaculum* sp. DTU813 and *Peptococcaceae* sp. DTU890 (26% of aggregate relative abundance). Although *Pelotomaculum* spp. are known syntrophic propionate-oxidizing *Bacteria*,^73^ no propionate was provided in the feed of the H_2_/CO_2_-fed reactor. The metabolic reconstruction indicated that *Methanothermobacter* sp. DTU779 could produce pyruvate, suggesting a survival strategy of *Pelotomaculum* sp. DTU813, based on a parasitic relationship with *Archaea* in this specific condition. The flux balance analysis performed on this reactor actually revealed that the couple has a commensalistic behavior, with *Pelotomaculum* sp. DTU813 being favored by the coexistence. *Methanothermobacter* sp. DTU779 is not indeed negatively influenced by the coexistence, thus explaining its high abundance in the community (Table S8). This hypothesis, based on the gene content and metabolites presence, was also supported by previous literature. In fact, *Pelotomaculum thermopropionicum* is known for fermenting pyruvate into acetate and propionate (3:1 molar ratio)^73^ and the same products were measured in the reactor (Figure 5). Finally, acetate, potentially produced by *Pelotomaculum* sp. DTU813, could be further utilized by *Peptococcaceae* sp. DTU890 for biomass production, with the consequent CO_2_/H_2_ generation. However, *Peptococcaceae* sp. DTU890 seems to have a versatile metabolism that can alternatively produce or consume different carbon sources (*i.e.*, acetate and CO_2_/H_2_) depending on the metabolites’ concentrations in the medium.

### 3.3 Proposed Mechanisms for Ammonia Acclimatization

The adaptation of microbiome to ammonia through the strategy of single and simple carbon source cultivation under stepwise increased ammonia levels achieved the specialized and simplified microbiome discussed above. Most importantly, it also clarified some aspects of the mechanisms involved in ammonia resistance by identifying the metabolic pathways involved in the adaptation and unraveling the trophic niches occupied by each MAG. The variable capabilities of different microbiomes to tolerate ammonia seemed to be connected with the homeostatic system, energy conservation strategies, and different ATP generation *via* substrate-level phosphorylation (Table S6).

**Figure 6:**
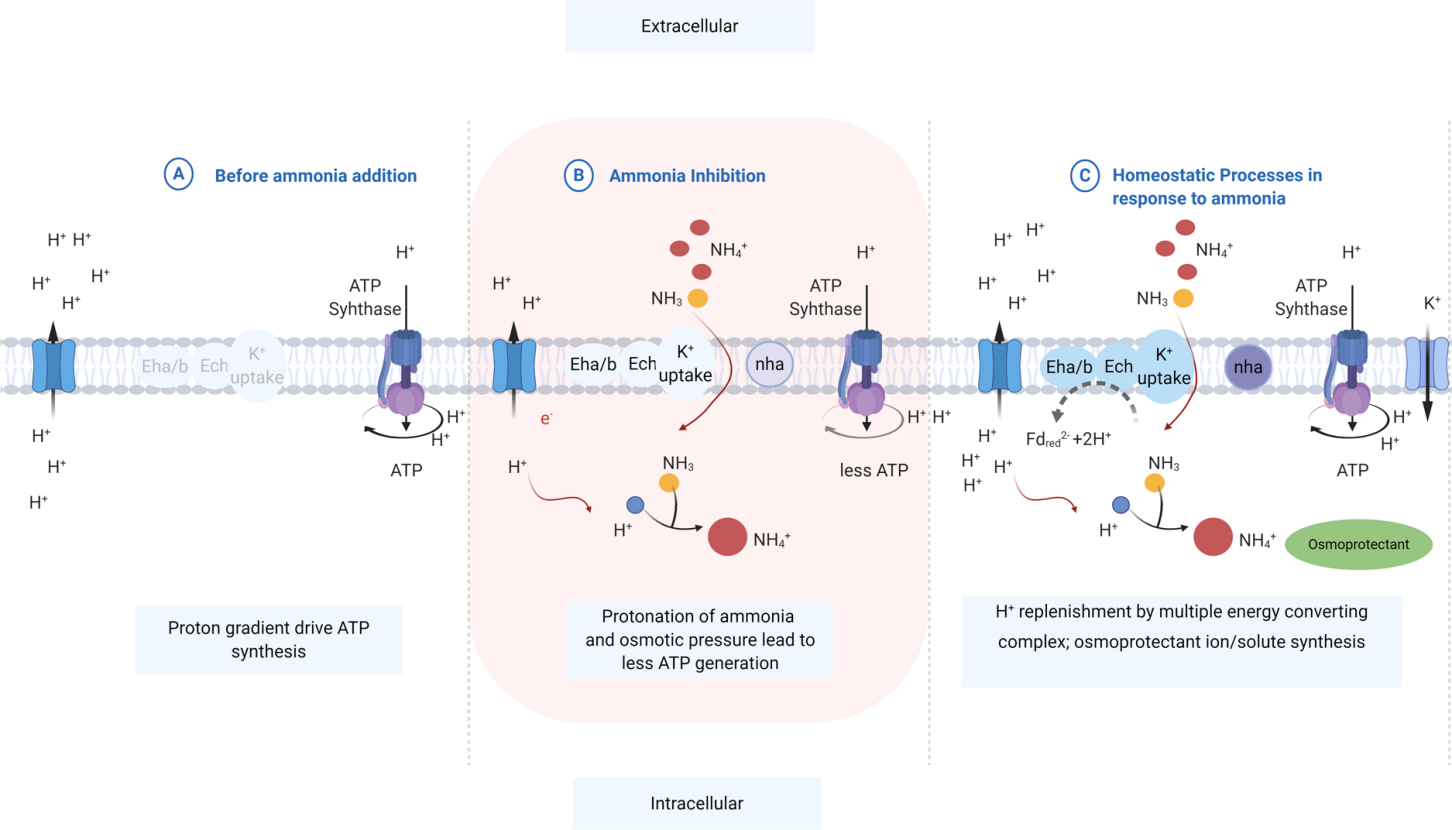
Proposed response of methanogen in different situations. (a) Before ammonia inhibition. (b) During ammonia inhibition, the protonation of ammonia and osmotic pressure lead to less ATP generation. (c) Homeostatic regulation in response to ammonia by following strategies: H^+^ replenishment by multiple energy-converting complexes, osmoprotectant ion/solute synthesis, and H^+^ binding by pumping K^+^ into the cell for cation balance.

From the homeostatic perspective, the presence of the potassium or sodium/proton antiporter (nha) system and the K^+^ uptake system (TrKA) had the potential to top-up intracellular protons and K^+^ for homeostatic processes, including the regulation of the turgor pressure and maintenance of cytoplasmic pH in response to the protonation of ammonia (Figures 2–6 and Table S6). As a confirmation of this process, Kraegeloh *et al.* revealed a process in which the loss of TrKA abolished any K^+^ uptake activity leading to osmotic sensitivity.^7^ Considering the osmotic stress induced by ammonium, a possible resistance mechanism of the MAGs identified in the current study could be related to the activity of glutamate dehydrogenase, glutamine, glycine betaine, and N^ε^-acetyl-l-lysine synthase. These enzymes can synthesize known osmoprotectants such as glutamate, glutamine, glycine betaine, and N^ε^-acetyl-l-lysine, which contribute to the survival of the cells at high osmotic stress and allow the colonization of ecological niches in severe environmental conditions. It seemed that the co-occurrence of the two systems (*i.e.*, osmoprotectant generation and potassium uptake) was a necessity against ammonia stress. This finding was in agreement with previous studies, highlighting that the synthesis of glutamate requires a stable level of K^+^.^74−76^

To regulate proton balance, potassium uptake, and biosynthesis maintenance, extra energy is needed. Thus, the raised question is how energy conservation can be achieved in order to survive during ammonia inhibition. The metabolic reconstruction provided novel insights regarding membrane-bound NADH-ubiquinone oxidoreductase (Nuo) in *Methanomassiliicoccales* sp. DTU777. In fact, the presence of a Fpo-like complex capable of reoxidizing the reduced ferredoxin, with simultaneous translocation of protons or sodium ions across the membrane, can generate the proton gradient needed for the ATP synthesis, as previously reported.^12,64^ Similarly, Hdr, Fwd, and Fdh present in *Methanothermobacter* sp. DTU779 were described to support the assembly of a bifurcating multienzyme complex, and Mnh was employed as an electrochemical potential-driven transporter (Figure 5 and Table S6).

Additionally, the coexistence of Eha/b and Ech complexes, in the presence of optimized energy conservation in DTU779, could be a reason for its extraordinary adaption to ammonia-inhibiting conditions (Figure 5). This hypothesis may be supported by the ability of *Methanothermobacter* sp. to outcompete other methanogens for establishing a syntrophic relationship with fatty acid-oxidizing *Bacteria*.^16^ Interestingly, the genome comparison of three identified *Archaea* in this study (*Methanoculleus* sp. DTU886, *Methanomassiliicoccales* sp. DTU777, and *Methanothermobacter* sp. DTU779) with the four ammonia-sensitive *Methanosaeta* spp. (downloaded from public databases) verified that the Eha/b and Ech energy-converting system was only present in the former three methanogens.

Obviously, when exposed to ammonia stress, methanogens with the multiple energy-converting hydrogenases mentioned above could become more energy-efficient and thereafter thrive easier than methanogens without these complexes (Figure 6). Additionally, the number of genes responsible for energy conservation in *Methanothermobacter* sp. DTU779 (n:25) was much higher than in *Methanomassiliicoccales* sp. DTU777 (n:18) and *Methanoculleus* sp. DTU886 (n:13), which is consistent with the variable capability of ammonia tolerance of each methanogen (Table S6).

Differential tolerance to ammonia might also be attributed to variable Gibbs free energies obtained by the different microbes from substrate-level phosphorylation. According to previous studies (listed in Table 1), the energy for cell maintenance could be obtained *via* methanogenesis from methanol (−315 kJ/per reaction),^20^ H_2_/CO_2_ (−135.6 kJ/per reaction),^77^ formate (−130.4 kJ/per reaction),^77^ and acetate (−36 kJ/per reaction).^21^ Obviously, methanogenesis from methanol and H_2_/CO_2_ is far more exergonic compared to the other methanogenic processes, which might lead to the higher ammonia tolerance of *Methanomassiliicoccales* sp. DTU777 in *G*_methanol_ and *Methanothermobacter* sp. DTU779 in *G*_H_2_/CO_2__ than *Methanoculleus* sp. DTU886 in *G*_acetate_. In particular, it is known from the literature that the conversion of 1 mol methanol to acetate in *Clostridiales* sp. and *Syntrophaceticus* sp. could release 0.625 ATP (the highest ATP gain identified for acetogens so far), with efficient sustained cell growth at energy-limited situations.^24,64^ Interestingly, in *G*_formate_, *Methanothermobacter* sp. DTU779 could only grow at an ammonia level up to 5.25 N–NH^+^ g/L, whereas it could stand up to 7.25 N–NH^+^ g/L in H/CO feeding, aided by the cooperative interaction with *Pelotomaculum* sp. DTU813. Furthermore, the presence of other intermediate metabolites (*e.g.*, acetate and propionate) in the four reactors indicated that alternative exergonic pathways were occurring. Specifically, the energy released from the conversion of acetate to propionate (−76.1 kJ/mol)^23^ and methanol to acetate (−71 kJ/mol)^24^ might support the growth of the whole consortium. Thus, ATP derived from *Archaea* and *Bacteria via* substrate-level phosphorylation might play a crucial role in overcoming bioenergetic barriers induced by ammonia inhibition and in driving thermodynamically unfavorable reactions. Besides, the difference in net ATP gain among the four microbial groups (*G*_acetate_, *G*_methanol_, *G*_formate_, and *G*_H_2_/CO_2__) might determine the variable capabilities of ammonia tolerance.

## Supporting Information Available

BA medium composition, characteristics of the reactors, and methane yields in the four groups; details and statistics for the total MAGs; average nucleotide identity comparison with previous projects; statistics and relative abundances of the MAGs selected according to MIMAG quality; KO terms used in KEGG mapper to visualize the metabolic pathways per MAG; metabolic reconstruction of the dominant MAGs; diversity indexes calculated for each sample using Nonpareil; results of pairwise interactivity in the microbiome of the four reactors characterized by different carbon sources (XLSX)
